# Points mean prizes: priority points, preferential status and directed organ donation in Israel

**DOI:** 10.1186/2045-4015-3-8

**Published:** 2014-02-24

**Authors:** Antonia J Cronin

**Affiliations:** 1 NIHR Biomedical Research Centre, Guy’s and St. Thomas’ NHS Foundation Trust and MRC Centre for Transplantation, King’s College, London, 5th Floor Tower Wing, Guy’s Hospital, Great Maze Pond, London SE1 9RT, UK

## Abstract

The introduction of Israel’s new Organ Transplantation Act in 2010 has enabled the development of a unique priority point system aimed at motivating individual’s to donate their organ. The priority point system rewards those who are willing to donate an organ with preferential status and an increased chance of receiving a donor organ, should they come to be in need of one. Preliminary evidence suggests it has considerable public support among Israelis, who appear willing to redress the challenge posed by those who are willing to accept an organ but not willing to donate. Since the Act’s introduction Israel has witnessed record numbers signing donor cards and there has been a significant increase in the actual numbers of transplants.

One aspect of the new Israeli system that has hitherto not much been considered is its tendency towards a communitarian model of organ donation and the implications this change in emphasis may have for the existing ‘opt-in’ model based upon autonomy and consent. Gil Siegel draws our attention to this aspect when he sets out his defence of a proposal he refers to as ‘directed organ donation to other registered donors’, which encourages community responsibility without affecting the established commitment to consent and individual freedom.

This commentary provides a brief overview of the new Act and its priority point system. It also examines Siegel’s proposal and considers the implications it may have for equity and justice, personal choice and dispositional authority. It is argued that although the proposal brings with it several inevitable hurdles for policy makers these are not insurmountable. Rather, its extraordinary potential to save life and avoid suffering should prompt urgent action at policy level. If such a scheme was successfully implemented in Israel it would represent a landmark change in organ donation and allocation policy, and set an example from which we all could learn.

## Introduction: the Organ Transplant Act 2008

In January 2010 a new law governing organ donation and allocation, the Organ Transplant Act 2008, came into effect in Israel [1]. The adoption of this pioneering Act was prompted by the shortage of organs available for transplantation. At the time Israel’s rates of organ donation were one of the lowest among developed countries [2].

The Act’s principal aim is to increase the number of deceased donor organ donations. In order to achieve this, it introduces a priority point system, set out at policy level, intended to motivate individuals to donate their organs [3]. According to the system, a person can gain priority points by signing a donor card, making a non-directed/non-specified organ donation during their lifetime, or as a result of a first-degree relative signing a donor card or consenting to procurement of organs after death. The resulting tiered system includes the following: (a) maximum priority; (b) regular priority; and (c) second priority. Maximum priority is granted to candidates if (a) consent has been given for organ donation from a deceased first-degree relative or (b) they donated a kidney, a liver lobe, or a lung lobe in the course of their life to a non-specified recipient. Regular priority is granted to candidates who hold a donor card; that is, those who have consented to donate their organs after their death. Second priority is granted to candidates with a first-degree relative who holds a donor card, even if they do not hold a donor card themselves [1,3,4].

The priority point system rewards those who are willing to donate an organ with preferential status and an increased chance of receiving a donor organ, should they come to be in need of one. The system is consistent with the view that since the supply of donor organs has been outstripped by demand, and they may be considered a scarce societal resource, a fair concept of justice demands that those who are willing to receive an organ should also be willing to donate one. It also redresses the perceived unfairness of ‘free riders’ willing to receive an organ but unwilling to donate.

The Israeli priority point system is unique. There are aspects of the system that have been subject to criticism, for instance the perceived unfairness of aligning the moral good of living donation with the expression of intent to donate. There has also been concern raised regarding the system’s apparent failure to allocate points to living donors who have directed their donation to a loved one, their child say, and its potential susceptibility to strategic behaviour [1,4]. But, overall, the system has been remarkably well received. As a matter of fact, since its introduction Israel has witnessed record numbers signing donor cards and there has been a significant increase in the actual numbers of transplants [2,4].

## Making the case for directed donation to registered donors

One aspect of the new Israeli system that has hitherto not much been considered is its tendency towards a communitarian model of organ donation and the implications this change in emphasis may have for the existing ‘opt-in’ model based upon autonomy and consent.

It is this aspect that Gil Siegel draws our attention to in his article *Making the case for directed organ donation to registered donors in Israel*[5]. Siegel claims that because the majority of the Israeli public supports organ donation and its proven benefits, organ recovery policy should be grounded in a strong communitarian strategy, as we all stand to benefit from cooperation. The challenge, he says, is to “design a policy that will increase the supply of organs without significant secondary harms, such as creating social segregation, increasing discrimination, or engaging in unethical conducts such as coerced retrieval” [5].

By setting out empirical evidence and normative arguments Siegel develops an eloquent defence of a proposal which encourages community responsibility without affecting the established commitment to consent and personal choice; he refers to the proposal as “directed organ donation to other registered donors” (DDRD). This, he says, is a scheme where individual preferences that promote just sharing of the burden (donating organs) as well as the benefits (receiving an organ) of transplantation medicine are respected. DDRD, he goes on, reinforces the idea of ‘reciprocal altruism’ and should be understood and portrayed as a transitional step towards a more communitarian model, as a signal of solidarity by sharing organs as a public good rather than as an exercise of a quasi-property right. It is noteworthy that a similar scheme has long since been promoted by a non-governmental organization in the US, but with only limited success [6]. It is also important to differentiate DDRD from the ‘preferred status’ set out in the priority point system. In DDRD the locus of the dispositional authority and allocation decision lies with the donor, not the official allocation body; priority points are not the decisive factor; and the overall benefit is collective rather than individual, insofar as it is available to all who sign up to be donors.

Siegel’s evidence includes a report on the findings from a detailed telephone survey, which included an assessment of Israeli attitudes towards DDRD. Interestingly a significant proportion (64%) of the sample felt that DDRD is justified, though remarkably, support was significantly higher in the Arab group (84%), and lower in the ultraorthodox Jewish group (50%). Moreover, the possibility of DDRD had a positive effect insofar as all groups sampled reported that the likelihood of their willingness to sign a donor card would ‘greatly increase’ if such a scheme were in place [5].

Siegel’s normative account and the overwhelming public support for DDRD demonstrated by the telephone survey findings suggest, from a utilitarian perspective at least, a ‘win win’ situation. Indeed, on the basis of survey findings alone, one might conclude the possibility of implementing a DDRD scheme in Israel should be taken seriously.

However, there are two further issues that merit careful consideration:

First, the dominant global trend in deceased donor organ allocation systems is that organs should be allocated according to principles of equity and justice, with the emphasis upon those with the greatest medical need. The proposed DDRD scheme moves away from this emphasis. If such a scheme were permissible, the principles of equity and justice would inevitably be compromised in specific instances [7,8]. Siegel considers the arguments and ethical objections to DDRD based upon the allocation of organs solely on objective medical criteria. One objection, Siegel says, citing Saunders, is that such allocation “reflects a perception of an altruistic donation and allocation system where the donor is permitting social agents (allocation committees) to administer just distribution of a scarce collective good” [9]. Since, the objection goes on, DDRD introduces a motivating factor and restricts access to those who do not participate, it could be regarded as harming altruism. In response to this objection, Siegel says it is almost impossible to assess ‘altruism’ and that it is not clear that DD is actually harming altruism [5]. But the evidence from the telephone survey that Siegel himself presents suggests that it is possible to provide an assessment of altruistic voluntariness. Moreover, and contrary to his concern, when we recall that all groups sampled reported that the likelihood of their willingness to sign a donor card would ‘greatly increase’ if such a scheme were in place, it seems pretty clear that DDRD would not harm it.

We must not however lose sight of what matters here. What matters is fairness and equality of opportunity to those in greatest need. The concerns of those who claim directed donation is discriminatory and may result in social segregation need to be taken seriously. Although Siegel’s proposed DDRD scheme is well intended, the only way in which policy could ensure that it would not, under any circumstances, compromise principles of equity and justice would be to compel every one of us to register as a donor. In that event, upon our death our organs, provided they were suitable, would be routinely removed and put to good use to benefit another registered donor, and so on. A policy proposal such as this would obviate the need to rely on altruism with its inherent limitations, and do away completely with ‘free riders’ [10].

Such consideration leads to the second issue, which concerns individual choice, autonomy, and dispositional authority. As Siegel explains, individual choice and autonomy have come to occupy an important role in healthcare ethics and law [5]. They take centre stage in ‘opt-in’ models of deceased organ donation. As such, a model of organ donation based upon compulsion, its good effects notwithstanding, is highly unlikely ever to be incorporated into policy. What we must therefore turn our attention to and question is how an individual’s choice to donate the ‘gift of life’ transforms into a public resource? If organs are to be considered as public resources to be distributed by relevant agencies, then from where did such dispositional authority arise? Why should those who donate their organs to the deceased donor pool not maintain the authority that entitled others to remove their organs in the first place? Living donors, in the context of a pre-existing relationship of some kind or another, maintain such authority [7].

Siegel’s telephone survey findings set out above provide food for thought in this regard, and suggest that an individual’s dispositional authority over their organs is accorded considerable importance among Israelis. So, if we continue to accede to a model of deceased organ donation based upon individual choice and authorization, we must provide good reason why such authorization does not allow for the possibility of individual deceased donors placing restrictions or conditions upon such authorization before their death, including the condition that an individual’s donation is directed to another registered donor. But Siegel’s findings go further and give us some insight into the sorts of directedness that matter to Israelis. He reports that 81% of those sampled (92% of Arab and 72% of ultraorthodox Jewish respondents) found directed donation to a family member in need, so-called partial directed donation, was justified [5]. What this highlights is that although willingness to support a communitarian strategy through directed donation is remarkably high among Israelis, willingness to support family, perhaps unsurprisingly, is even greater. Although the likelihood of an individual donating their organs to a family member in the event of their death is low, strategic policy aimed at encouraging a communitarian approach to organ donation by allowing DDRD must take this possibility into account. Moreover, if policy is seen to encourage directedness of one sort, it must provide robust reasons for restricting directedness of other sorts, lest the concerns of those who claim directed donation is discriminatory and may result in social segregation become ever more germane.

## Conclusion

Israel’s new Organ Transplantation Act has enabled the development of a unique priority point system aimed at motivating individuals to donate their organs. Although the system is not ideal and has not yet reached maturity, it has set a remarkable precedent, and experience gained from it will undoubtedly become a useful resource. Preliminary evidence suggests it has considerable public support among Israelis who appear willing to redress the challenge posed by those who are willing to accept an organ but not willing to donate.

Siegel’s DDRD proposal embraces this willingness and encourages community responsibility without curtailing individual freedom or liberty. Though it brings with it several inevitable hurdles for policy makers these are not insurmountable. Rather, its extraordinary potential to save life and avoid suffering should prompt urgent action at policy level. If the DDRD scheme is successfully implemented in Israel it would represent a landmark change in organ donation and allocation policy, and set an example from which we all could learn.

## Competing interests

The author declares that she has no competing interests.

## Author information

Dr. Antonia J Cronin is a Consultant Nephrologist and Honorary Senior Lecturer at Guy’s and St. Thomas’ NHS Foundation Trust and the MRC Centre for Transplantation at King’s College, London. She also holds a Master’s degree and PhD in Medical Law and Ethics. She is currently principal investigator on a policy impact research project entitled ‘Increasing understanding on the quality and allocation of organs for transplants’, funded by King’s Policy Institute at King’s College, London.

## “Commentary on” statement

Commentary on Siegel G, ‘Making the case for directed organ donation to registered donors in Israel’ *Israel Journal of Health Policy Research* 2014; 3:1.

## References

[B1] Lavee J Ashkenazi T Gurman G A new law for allocation of donor organs in Israel Lancet 2010 375 1131 10.1016/S0140-6736(09)61795-5 20022099

[B2] Figures http://www.irodat.org accessed 03 February 2014

[B3] Organ Transplantation Law 5768–2008, Israeli Book of Laws (English Translation provided by the Israeli Ministry of Justice). Section 9(b)4 of the Act authorizes the Steering Committee for the National Transplant Centre to “draw up directives in the matter of the allocation of organs” http://old.justice.gov.il/NR/rdonlyres/F8E6E7B8-D67A-475A-B64A-F9B86797F282/9952/2144.pdf

[B4] Quigley M Wright L Ravitsky V Organ donation & priority points in Israel: an ethical analysis Transplantation 2012 93 970 973 10.1097/TP.0b013e31824e3d95 22461040

[B5] Siegel G Making the case for directed organ donation to registered donors in Israel Isr J Health Policy Res 2014 3 1 10.1186/2045-4015-3-1 24456997 PMC3909280

[B6] Undis DJ LifeSharers: increasing organ supply through directed donation Am J Bioethics 2005 5 22 24 10.1080/15265160500194600 16109688

[B7] Cronin AJ Price D Directed organ donation: is the donor the owner? Clin Ethics 2008 3 127 131 The arguments here are complex and include consideration of whether the body and its parts can be considered property 10.1258/ce.2008.008018 20890462 PMC2948558

[B8] Cronin AJ Douglas JF Directed and conditional organ donation: laws and misconceptions Med Law Rev 2010 18 3 275 301 10.1093/medlaw/fwq019 20739435 PMC2927926

[B9] Saunders B Altruism or solidarity? The motives for organ donation and two proposals Bioethics 2012 27 380 10.1111/j.1467-8519.2012.01989.x 22827319

[B10] Cronin AJ Harris J Authorisation, altruism and compulsion in the organ donation debate J Med Ethics 2010 36 627 10.1136/jme.2009.031245 20880894

